# Implementation and security analysis of practical quantum secure direct communication

**DOI:** 10.1038/s41377-019-0132-3

**Published:** 2019-02-06

**Authors:** Ruoyang Qi, Zhen Sun, Zaisheng Lin, Penghao Niu, Wentao Hao, Liyuan Song, Qin Huang, Jiancun Gao, Liuguo Yin, Gui-Lu Long

**Affiliations:** 1 State Key Laboratory of Low-Dimensional Quantum Physics and Department of Physics, Tsinghua University, Beijing, 100084 China; 2 School of Information and Technology, Tsinghua University, Beijing, 100084 China; 3 Beijing National Research Center for Information Science and Technology, Beijing, 100084 China; 4 School of Electronic and Information Engineering, Beihang University, Beijing, 100191 China; 5 Innovative Center of Quantum Matter, Beijing, 100084 China; 6 Beijing Academy of Quantum Information Science, Beijing, 100193 China

**Keywords:** Fibre optics and optical communications, Quantum optics

## Abstract

Rapid development of supercomputers and the prospect of quantum computers are posing increasingly serious threats to the security of communication. Using the principles of quantum mechanics, quantum communication offers provable security of communication and is a promising solution to counter such threats. Quantum secure direct communication (QSDC) is one important branch of quantum communication. In contrast to other branches of quantum communication, it transmits secret information directly. Recently, remarkable progress has been made in proof-of-principle experimental demonstrations of QSDC. However, it remains a technical feat to bring QSDC into a practical application. Here, we report the implementation of a practical quantum secure communication system. The security is analyzed in the Wyner wiretap channel theory. The system uses a coding scheme of concatenation of low-density parity-check (LDPC) codes and works in a regime with a realistic environment of high noise and high loss. The present system operates with a repetition rate of 1 MHz at a distance of 1.5 kilometers. The secure communication rate is 50 bps, sufficient to effectively send text messages and reasonably sized files of images and sounds.

## Quantum communication: Security despite noise

A quantum communication system demonstrated by researchers in China can transfer information securely in a realistic noisy environment. Emerging supercomputers and quantum computers may soon break the classical encryption methods that protect our information, highlighting the need for new cryptographic techniques based on quantum mechanics. Gui-Lu Long at Tsinghua University, Beijing, and co-workers have demonstrated a form of quantum secure direct communication (QSDC) that transfers information directly without the need to distribute keys, which are vulnerable to attacks. The team used a laser to generate single photons, which could carry secure quantum information such as text messages and image files over a distance of 1.5 kilometers. The information was decoded successfully by the receiver, even when the situation was made realistic by causing high photon loss or introducing errors due to noise.

## Introduction

Economic, political, and social well-being in the world depend crucially on secure communication infrastructures. Present communication is secured through encryption techniques, relying on pre-shared key and cryptographic protocols built on the computational difficulty of certain mathematical problems, for example, the RSA public key scheme^1^. There are potential dangers with the present secure communication system. On one hand, these cryptographic protocols are based on mathematically difficult problems that are not rigorously proven to have no efficient solution algorithms. These protocols may be broken one day, or might have been broken privately already by some genius; we do not yet know whether efficient algorithms for solving these problems exist. On the other hand, some cryptography may become insecure with the rapid development of supercomputers and the prospect of practical quantum computers^2^. In contrast to cryptographic algorithms, physical-layer security is based on the conditions that the eavesdropper has unlimited computing power, but the legitimate receiver has a physical advantage over the eavesdropper. In 1975, Wyner presented a degraded wiretap channel model^3^, which is a basic channel model when security is concerned. Secrecy capacity is defined as the supremum of all the achievable transmission rates with security and reliability. For classical communication, estimation of the secrecy capacity in a practical communication system is hard, because it is difficult for the legitimate parties to detect eavesdropping. When quantum systems such as single photons or entangled pairs of photons are used to transmit digital information, quantum physics principles give rise to novel capability unachievable with classical transmission media^4^. It is impossible in principle for Eve to eavesdrop without disturbing the transmission so as to avoid detection. The first quantum communication protocol, proposed by Bennett and Brassard (BB84)^5^, showed how to exploit quantum resources for secure key agreement. Quantum-key distribution^5–9^ distributes a random key, rather than the information itself, and the information is sent through another classical communication channel.

In 2000, quantum secure direct communication (QSDC) was proposed^10^. QSDC can communicate information directly without key distribution^10–14^, which eliminates further security loopholes associated with key storage and ciphertext attacks^15,16^, offering a new tool for selection in the zoo of secure communication protocols. Recently, experiments were completed of proof-of-principle demonstrations of QSDC based on single photons^17^ and entangled pairs^18,19^. In particular, Zhang et al.^19^ demonstrated QSDC in a fiber over a meaningful distance of 500 m using the two-step QSDC protocols^10,11^.

Here, we report an experimental implementation of a practical quantum secure communication system using a protocol based on the DL04 protocol^12^. To move QSDC forward into practical application, a number of key issues must be solved. Security analysis of information transmission is crucial for practical application. According to Wyner’s wiretap model, it is essential to let the system work at a capacity below the secrecy capacity of the channel. We estimated the secrecy capacity using the error rate from the sampling-checking process of the system. Once this secrecy capacity estimation is completed, it is possible to design a coding scheme with a communication rate smaller than this secrecy capacity. We have developed a coding scheme using concatenation of low-density parity check (LDPC) codes^20,21^. The scheme is specifically designed for operating in the high loss and high error-rate regime, unique for quantum communication. The experiment shows that our QSDC platform can work effectively in a realistic environment. In our system, the single-photon source was an attenuated faint laser pulse with a repetition rate of 1 MHz. The distance was 1.5 km, and the secure information transmission rate achieved was 50 bps, sufficient to transmit text messages and image or sound files of reasonable size.

## Results

### Practical DL04-QSDC (PDL04 QSDC) protocol

**Fig. 1:**
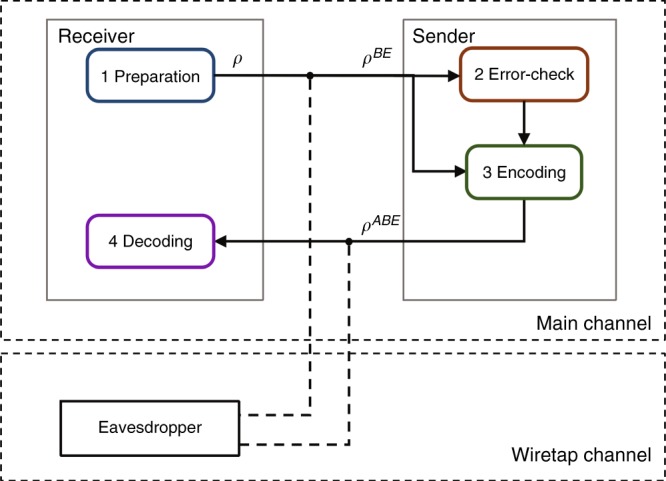
Illustration of the PDL04-QSDC protocol. The “main channel” and the “wiretap channel” are discrete memoryless channels. The main channel represents a channel between the sender and the legitimate receiver, while the wiretap channel represents a channel between the sender and the eavesdropper

Our practical quantum secure direct communication scheme is based on the DL04 protocol using single photons^12^. The scheme is illustrated in detail in Fig. 1. The “main channel” and the “wiretap channel” are discrete memoryless channels; the main channel represents the channel between the sender and receiver, while the wiretap channel represents the channel between the legitimate users and the eavesdropper. The protocol contains the following four steps.

1. Bob, a legitimate information receiver, prepares a sequence of qubits. Each qubit is randomly in one of the four states $\left| 0 \right\rangle$, $\left| 1 \right\rangle$, $\left| + \right\rangle$, and $\left| - \right\rangle$, where $\left| 0 \right\rangle$, $\left| 1 \right\rangle$ are the eigenstates of Pauli operator *Z*, and $\left| + \right\rangle$, $\left| - \right\rangle$ are the eigenstates of Pauli operator *X*. Then, he sends the sequence of states to the information sender Alice.
2. After receiving the single photon sequence, Alice randomly chooses some of them and measures them randomly in the *Z*-basis or the *X*-basis. She publishes the positions, the measuring basis and measurement results of those single photons. Bob compares this information with his preparations of these states, estimates the bit-error rate of the Bob-to-Alice channel, and informs Alice through a broadcast channel. Thus, Alice can estimate the maximum secrecy capacity *C*_*s*_ of the Bob-to-Alice channel using the wiretap channel theory.
3. Alice chooses a coding scheme for the remaining qubits. This coding scheme is based on the concatenation of LDPC codes that will be described in the discussion section. The following two unitary operations,
  $$I = \left| 0 \right\rangle \left\langle 0 \right| + \left| 1 \right\rangle \left\langle 1 \right|,Y = \left| 1 \right\rangle \left\langle 0 \right| - \left| 0 \right\rangle \left\langle 1 \right|$$
  map ‘0’ and ‘1’, respectively; they are further used for constructing the code words. Then, she sends them back to Bob.
4. Bob decodes Alice’s message from his received signals after measuring the qubits in the same basis he prepared them. If the error rate is below the correcting capability of the LDPC code, the transmission is successful. Then, they start again from step (1) to send another part of the secret message until they complete the transmission of the whole message. If the error rate is larger than the correcting capability of the LDPC code, neither Bob nor Eve can obtain information. In this case, they terminate the process.

### Security analysis

According to Wyner’s wiretap channel theory^3^, the secrecy capacity is

1 $$C_s = \mathop {{\max }}\limits_{\{ p\} } \left\{ {I(A:B) - I(A:E)} \right\}$$

where *p* represents the probability of unitary operation *I*. *I*(*A*:*B*) and *I*(*A*:*E*) are the mutual information between Alice and Bob and between Alice and Eve, respectively. Moreover, *I*(*A*:*E*) represents the maximum information that an eavesdropper can obtain using the best strategy she can.

The state Bob prepared is a complete mixed state, $\rho = \left( {\left| 0 \right\rangle \left\langle 0 \right| + \left| 1 \right\rangle \left\langle 1 \right|} \right)/2$, because he prepares it with equal probabilities of the four states, $\left| 0 \right\rangle$, $\left| 1 \right\rangle$, $\left| + \right\rangle$, $\left| - \right\rangle$. We consider the case of collective attack, where the most general quantum operation that Eve may perform in the forward Bob-to-Alice channel consists of a joint operation on the qubit and some ancilla that belong to Eve,

2 $$\rho ^{BE} = U\left( {\rho \otimes \left| \varepsilon \right\rangle \left\langle \varepsilon \right|} \right)U^ + $$

where $\left| \varepsilon \right\rangle$ represents Eve’s ancillary state and *U* is a unitary operation acting on the joint space of the ancilla and the qubit. Then, Eve resends the qubit to Alice and stores her ancilla until the qubit is sent back. Alice performs an operation*I*with probability *p* or *Y* with probability 1−*p*. After operating by Alice, the state becomes

3 $$\rho ^{ABE} = p \cdot \rho _0^{BE} + \left( {1 - p} \right) \cdot \rho _1^{BE}$$

where $\rho _0^{BE} = I\rho ^{BE}I$ and $\rho _1^{BE} = Y\rho ^{BE}Y^ +$. To gain Alice’s information, Eve must distinguish Alice’s encoded qubit $\rho _0^{BE}$ from $\rho _1^{BE}$ by performing coherent measurements on any number of qubits and ancilla. The maximum mutual information between Alice and Eve is upper-bounded by:

4 $$I(A:E) \le \chi = \, \mathop {{\max }}\limits_{\{ U\} } \left\{ S(\rho ^{ABE}) - p \cdot S(\rho _0^{BE})\right. \\ - \left.(1 - p) \cdot S(\rho _1^{BE}) \right\}$$

where *S*(*ρ*) is the von Neumann entropy, and *χ* is the Holevo bound^22^. We obtain the maximum mutual information between Alice and Eve (the detailed derivation is given in supplementary information),

5 $$I(A:E) \le h(\xi )$$

where ${\xi = ( {1 - \sqrt {( {1 - 2p} )^2 + ( {1 - 2e_x - 2e_z} )^2[ {1 - ( {1 - 2p} )^2} ]} } )/2}$, *e*_*x*_ and *e*_*z*_ are the bit-error rates in the *X*-basis and the *Z*-basis in the error-check, respectively, and *h*(*x*) = −*x* log_2_ *x*−(1–*x*) log_2_ (1–*x*) is the binary Shannon entropy.

Because of imperfect efficiency of the detectors and channel loss, Bob cannot receive all the qubits. Gottesman has proven the security of the Bennet-Brassard quantum-key-distribution protocol in the case in which the source and detector are under the limited control of an adversary^23^. Similarly, considering the detectors and channel loss, the maximum mutual information between Alice and Eve becomes

6 $$I(A:E) \le Q^{{\rm Eve}} \cdot h(\xi )$$

where *Q*^Eve^ is the maximum rate at which Eve can access the qubits. Highly attenuated lasers are used as an approximate single-photon source in our implementation; for a better treatment of such an approximate single photon source, one can use the decoy state methods^24–26^.

The main channel can be modeled as a cascaded channel, which consists of a binary symmetric channel and a binary erasure channel in series^27^. The mutual information between Alice and Bob is,

7 $$I(A:B) = Q^{{\rm Bob}} \cdot \left[ {h\left( {p + e - 2pe} \right) - h(e)} \right]$$

where *Q*^Bob^ is the receipt rate at Bob’s side and *e* is the bit-error rate between Alice and Bob. We can estimate the lower bound of the secrecy capacity,

8 $$C_{s} = \mathop {{\max }}\limits_{\{ p\} } \left\{ {I(A:B) - I(A:E)} \right\}\\ = \mathop {{\max }}\limits_{\{ p\} } \left\{ {Q^{{\rm Bob}} \cdot \left[ {h\left( {p + e - 2pe} \right) - h(e)} \right] - Q^{{\rm Eve}} \cdot h(\xi )} \right\}\\ = Q^{{\rm Bob}} \cdot \left[ {1 - h(e)} \right] - Q^{{\rm Eve}} \cdot h\left( {e_x + e_z} \right)\\ = Q^{{\rm Bob}} \cdot \left[ {1 - h(e) - g \cdot h\left( {e_x + e_z} \right)} \right]$$

where *g* represents the gap between *Q*^Eve^ and *Q*^Bob^, depending on the back-channel loss and the efficiency of the detector.

For any wiretap channel, if the secrecy capacity is non-zero, i.e., if the legitimate receiver has a better channel than the eavesdropper, there exists some coding scheme that achieves perfect secrecy^3^. Not all coding schemes can guarantee the security; the security depends on the details of the coding.

### Experimental results

**Fig. 2:**
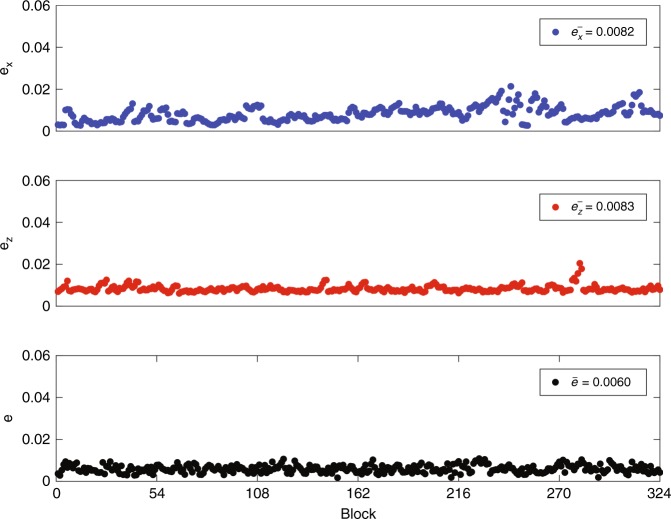
System stability with different message blocks. *e*_*x*_ and *e*_*z*_ are the error rates of measurements using the *X*-basis and *Z*-basis, respectively, at Alice’s site. *e* is the error rate at Bob’s site. We estimate the error rate block by block; each block contains 1312 × 830 pulses. The mean number of photons is 0.1. The inherent loss of a quantum channel is 14.5 dB, which includes the efficiency of the detector, ~70%, and the optical elements, ~13 dB. The total loss of the system is 25.1 dB at a distance of 1.5 km

**Fig. 3:**
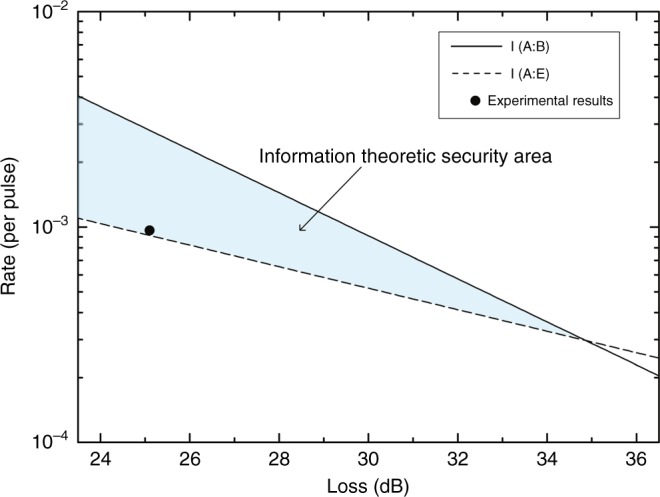
The solid line represents the mutual information between Alice and Bob, the capacity of the main channel that transmission rate cannot exceed, by the noisy-channel coding theorem. The dotted line is the mutual information between Alice and Eve, the maximum information that an eavesdropper can obtain. The error rates are set at values as above, namely *e* is 0.6% and *e*_*x*_ and *e*_*z*_ are 0.8%. Symbols represent experimental results. We set the length of the pseudo-random sequence as 830. Together with the chosen LDPC code, our coding scheme yields a transmission rate of 0.00096 when the bit-error rate is under 10^−6^. Because the rate is greater than the mutual information between Alice and Eve, both the security and reliability of the information transmission are assured

We implemented the above scheme in a fiber system with phase coding^28^. The details of the experimental setup and methods are shown in the material and methods section, and the coding scheme is described in the discussion section. In our experiment, we initially set the distance at 1.5 km, which is a typical distance between buildings in a secure area. Figure 2 shows the error rates at Alice’s and Bob’s sites; the horizontal axis is labeled with the number of blocks processed. *e*_*x*_ and *e*_*z*_ are the error rates of measurements using the *X*-basis and *Z*-basis at Alice’s site, respectively. We estimate the error rate block by block. Each block contains 1312 × 830 = 1,088,960 pulses, including a frame head for synchronization. Under normal working conditions, their values are ~0.8%. At Bob’s site, of the pulses he sent to Alice previously, he receives 0.3% of them; namely for every 1000 pulses, 3 photons are counted when Bob measures the returned pulses. The error rate at Bob’s site is lower than that at Alice’s site due to the intrinsic robustness of the retrace-structure of light, usually ~0.6%. Here, the mean photon number is 0.1. The inherent loss of the quantum channel is 14.5 dB, including the efficiency of the superconducting nanowire single-photon detectors, ~70%, and the optical elements, ~13 dB. Because the mean photon number is 0.1 and the channel loss of 1.5 km fiber is 0.6 dB, the total loss of the system is 25.1 dB. Shown in Fig. 3, the mutual information *I*(*A*:*B*) and *I*(*A*:*E*) versus the loss of the system are two straight lines. The area between these two lines is the information-theoretic secure area; i.e., for a coding scheme with an information rate within these areas, it is possible to guarantee the security reliably. In our experiment, the error rates are initially set at values as above, namely *e* is 0.6% and *e*_*x*_ and *e*_*z*_ are 0.8%. Then, the secrecy capacity is estimated as 0.00184 for loss at 25.1 dB. For the number *N* in the pseudo-random sequence, we set *N* = 830, after optimization. Together with the chosen error correcting code, our coding scheme gives a transmission rate 0.00096 when the bit error rate is chosen as 10^−6^. Additionally, $I(A:E) = g \cdot Q^{{\rm Bob}} \cdot h\left( {e_x + e_z} \right) = 9.1 \times 10^{ - 4}$, where the loss of the back channel, including the efficiency of the detector and channel loss, is ~4.1 dB, so that *g* = 2.57. This yields a secure information rate of 50 bps, which is well within the secure area in Fig. 3.

## Discussion

It is well-known that in quantum communication, photon loss is very high due to inefficient photon sources, high channel loss and low detector efficiency. To guarantee the reliability and security of transmission for QSDC, we designed a coding scheme based on the concatenation of LDPC codes, with preprocessing based on the universal hashing families (UHF)^29^.

**Fig. 4:**
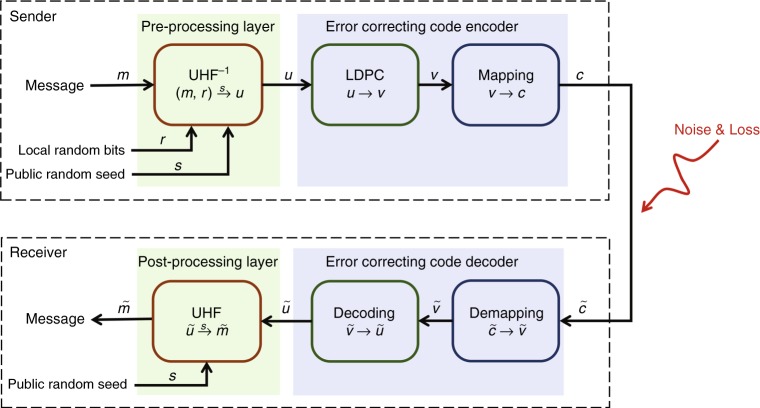
Illustration of the coding scheme. A message *m* together with a local random bits *r* and public random seed *s* are processed by the reverse universal hashing families UHF^−1^ to vector *u*, and then *u* is changed by LDPC code into *v*, which is mapped to codeword *c* and is then sent to the receiver's site. Because loss and error, receiver Bob receives a degraded codeword, and then he demaps, decodes and obtains the message after performing universal hashing families UHF

Details of our coding scheme are illustrated in Fig. 4. For each message block ***m*** of length *N*_*m*_, the sender, namely Alice, generates a local sequence of random bits, denoted ***r***, of length *N*_*r*_. Then, she maps (***m***, ***r***) to a vector ***u*** of length *N*_*u* _= *N*_*r* _+ *N*_*m*_, by the inverse of an appropriately chosen UHF, determined by a public random seed ***s***. Information theoretic security can be guaranteed if the ratio of the length of the random bits to the length of the code word is higher than the mutual information between Alice and Eve^30^. In information theory, the noisy-channel coding theorem establishes reliable communication for any given degree of noise contamination of a communication channel^31^. To ensure the reliability of the information, Alice encodes the vector ***u*** to ***v*** of length *N*_*v*_ using the generator matrix of a specified LDPC code. Then, she maps each coded bit to a sequence of length *N* to obtain a transmitted sequence, namely a code word of length *N*_*c*_ that is transmitted over the quantum channel. According to the noisy-channel coding theorem^31^, the ratio of the length of the vector ***u*** to the length of the code word cannot be higher than the channel capacity. We deduce that the information rate,

9 $$R = \frac{{N_m}}{{N_c}} = \frac{{N_u}}{{N_c}} - \frac{{N_r}}{{N_c}} \le I(A:B) - I(A:E) \le C_s$$

After receiving the modulated pulses from Alice, the legitimate receiver Bob makes measurements in the same basis as he prepared them. Though only a fraction of photons in a pseudo-random sequence can reach Bob’s site, he can still readout the coded bit by looking at the log-likelihood ratios of each coded bit calculated from the received sequence, and he decodes the LDPC code with an iterative propagation-decoding algorithm with the log-likelihood ratios. Then, Alice announces the public random seed ***s***, so that Bob can obtain the secure message by the certain UHF with the seed.

For our system, we consider a (1408, 1024) quasi-cyclic (QC)-LDPC code of block length *N*_*v*_ = 1408, which is a standardized LDPC code of the Consultative Committee for Space Data Systems (CCSDS) for use in near-earth and deep-space applications^32^. The last 128 coded bits in the obtained code word of this LDPC code are punctured to achieve better error-correction performance. Thus, the actual block length of punctured LDPC code word is reduced to 1280 and the actual code rate is 0.8. Then, each coded bit in the punctured LDPC code word is mapped into a pseudo-random sequence of length 830 to obtain a transmitted sequence of length *N*_*c*_ = 1280 × 830 = 1,062,400 such that our coding scheme has a transmission rate of 0.00096. During decoding, the log-likelihood ratio of each coded bit of LDPC code is first calculated based on its corresponding pseudo-random sequence. Then, an effective iterative propagation-decoding algorithm, the scaling Min-Sum decoding algorithm^33^, is used to decode this LDPC code. The maximum number of iterations and scaling factor of the scaling Min-Sum decoding algorithm are set to 65 and 0.75, respectively. This shows that the decoding bit-error rate is ~10^−6^ in our code scheme.

## Materials and methods

**Fig. 5:**
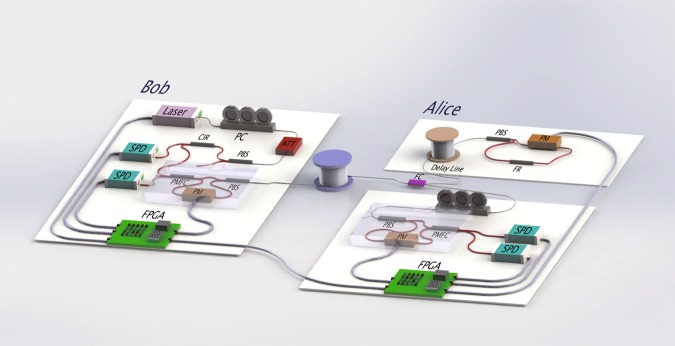
Experiment setup. A strongly attenuated 1550 nm laser is used as an approximate single-photon source with a systematic pulse-repetition frequency of 1 MHz. Bob sends the single photons to Alice in a superposition of two time-bins with a relative phase, and Alice randomly chooses one of two possible tasks, error-check or coding. Both sides are controlled by field programmable gate arrays (FPGAs), and the operation of the four single-photon states is realized with a commercial lithium niobate modulator. PM phase modulator. PC polarization controller. PBS polarization beam splitter. ATT attenuator. CIR optical circulator. FC fiber coupler. SPD superconducting nanowire single-photon detector with 70% detection efficiency, 100 Hz dark count rate and 50 ns reset time. PMFC polarization maintaining filter coupler. FR Faraday rotator

The experimental setup is shown in Fig. 5. Bob prepares a sequence of single-photon pulses. After polarization control and attenuation, the pulses go to the Mach-Zehnder ring in which a random phase of 0, π/2, π, and 3π/2, is encoded, which is equivalent to preparing qubits randomly in the $\left| 0 \right\rangle$, $\left( {\left| 0 \right\rangle + \left| 1 \right\rangle } \right)/\sqrt 2$, $\left| 1 \right\rangle$ and $\left( {\left| 0 \right\rangle - \left| 1 \right\rangle } \right)/\sqrt 2$ states, respectively. Then, it is sent to Alice’s site through a 1.5 km-long fiber. After arriving at Alice’s site, it is separated into two parts, one goes to the encoding module, and the other goes to the control module. In the control module, the qubits are measured, and the results are compared with Bob’s through the classical communication line connecting the two FPGAs shown at the bottom of Fig. 5. Simultaneously, encoding is performed in the encoding module. If the error rate is smaller than the threshold, the encoding part is allowed to send the single photons back to Bob through the same fiber; they then are guided to the single-photon detectors, where they are measured. The three phase modulators, the single photon detectors, and the encoding of messages are controlled at the two sites by the FPGAs, which are further controlled by upper-position computers.

The advantage of such forward-backward routing of the photon pulses is the automatic compensation of the drift of the polarizations of the time-bin pulses, because they exchange their routes after reflection by the Faraday rotator at Alice’s site. This automatic compensation design was proposed by Martilelli^34^ and has also been used in the plug-play QKD system^35^. The difference between the plug-play QKD scheme and DL04-based schemes, such as in refs. ^7,12,17^ and in this PDL04-QSDC scheme, is in the strength of light pulses in the forward channel. In refs. ^7,12,17^, single photons are used in both the forward and backward channels, whereas in plug-play QKD^35^, the forward channel uses strong classical light pulses; only the Alice-to-Bob backward channel uses single-photon pulses. This mechanism of automatic compensation of polarization fluctuation works both at the single photon level and at the strong-intensity level; hence, it greatly enhances the interference in our scheme and leads to high visibility^36^. However, in the check-module of our system, such a retrace-light circuit is not applicable, and active polarization compensation must be used; namely, one monitors the drift constantly and when it reaches some value, forcibly restores them. As a result, the error rate in the check mode is usually higher than that in the communication mode.

In summary, we have implemented a practical quantum secure direct-communication system in a realistic environment of high noise and high loss. To combat error and loss, LDPC code and pseudo-random sequence techniques are applied. The security of the system is analyzed in detail using the wiretap channel theory. Given the error rates, the secrecy capacity of the channel can be estimated. When the secrecy capacity is non-zero, a coding scheme with an information rate less than the secrecy capacity will ensure both the security of the information transmission and reliability of the information. At a practical meaningful distance of 1.5 km, a secure information rate of 50 bps is achieved. These parameters are premature, and there is much room for improvement. With current technology, an information rate of a dozens of kbps is achievable.

## Supplementary information

Supplementary Information
