# Supplementary material for: Implementation and security analysis of practical quantum secure direct communication
Source: Light Sci Appl. 2019 Feb 6;8:22. doi: 10.1038/s41377-019-0132-3 (PMC6363753; doi:10.1038/s41377-019-0132-3)
Supplement: Supplementary file 1 — Supplementary Information [file 41377_2019_132_MOESM1_ESM.docx]

**Supplementary Information to**

**Implementation and Security Analysis of Practical Quantum Secure Direct Communication**

Ruoyang Qi[[1]](#footnote-1),*, Zhen Sun^2,*^, Zaisheng Lin^2,*^, Penghao Niu^1^, Wentao Hao^2^, Liyuan Song^3^,

Qin Huang^3^, Jiancun Gao^1^, Liuguo Yin^2,4,†^, and Gui-Lu Long^1,4,5,6†^

The maximum mutual information between Alice and Eve can be calculated as follows

$$I(A:E)\leq\max_{\{U\}} \{S(\rho^{ABE})-p\cdot S(\rho_{0}^{BE})-(1-p)\cdot S(\rho_{1}^{BE})\}$$

where

$$\begin{matrix} \rho^{ABE}=p\cdot\rho_{0}^{BE}+(1-p)\cdot\rho_{1}^{BE} \\ =p\cdot U\left(\rho\otimes|\varepsilon\rangle\langle\varepsilon|\right)U^{+}+(1-p)\cdot YU\left(\rho\otimes|\varepsilon\rangle\langle\varepsilon|\right)U^{+}Y^{+} \end{matrix}$$

Since $\rho_{0}^{BE}$and $\rho_{1}^{BE}$ only differs from $\rho\otimes|\varepsilon\rangle\langle\varepsilon|$ by some unitary transformations,

$S(\rho_{0}^{BE})=S(\rho_{1}^{BE})=S(\rho)=1$.

Hence,

$I(A:E)\leq\max_{\{U\}} \{S(\rho^{ABE})\}-1$.

The effect of the unitary operation may be represented as

.

Unitarity is guaranteed if the following conditions are satisfied,

$\begin{matrix} \langle\varepsilon_{00}|\varepsilon_{00}\rangle+\langle\varepsilon_{01}|\varepsilon_{01}\rangle=1 \\ \langle\varepsilon_{11}|\varepsilon_{11}\rangle+\langle\varepsilon_{10}|\varepsilon_{10}\rangle=1 \\ \langle\varepsilon_{00}|\varepsilon_{10}\rangle+\langle\varepsilon_{01}|\varepsilon_{11}\rangle=0 \end{matrix}$.

The corresponding Gram matrix of $\rho^{ABE}$ is explicitly written as [1],

$$G=\frac{1}{2}\left(\begin{matrix} p & 0 & 2i\alpha\sqrt{p(1-p)} & \sqrt{p(1-p)}\delta\\ 0 & p & -\sqrt{p(1-p)}\delta^{*} & -2i\beta\sqrt{p(1-p)} \\ -2i\alpha\sqrt{p(1-p)} & -\sqrt{p(1-p)}\delta& 1-p & 0 \\ \sqrt{p(1-p)}\delta^{*} & 2i\beta\sqrt{p(1-p)} & 0 & 1-p \end{matrix}\right)$$

where$\alpha=\mathrm{Im}(\langle\varepsilon_{01}|\varepsilon_{00}\rangle)$, $\beta=\mathrm{Im}(\langle\varepsilon_{10}|\varepsilon_{11}\rangle)$, $\delta\text{=}\langle\varepsilon_{01}|\varepsilon_{10}\rangle-\langle\varepsilon_{00}|\varepsilon_{11}\rangle$.

The eigenvalues of G are given by

$$\lambda=\frac{1}{4}\pm\frac{1}{2}\sqrt{p(1-p)(\Delta_{1}\pm\Delta_{2})^{2}+(p-\frac{1}{2})^{2}}$$

where $\Delta_{1}\text{=}\sqrt{(\alpha-\beta)^{2}}$, $\Delta_{2}\text{=}\sqrt{(\alpha+\beta)^{2}+\delta\delta^{*}}$.

$$\begin{matrix} U|+\rangle|\varepsilon\rangle=\frac{1}{\sqrt{2}}\left(|0\rangle|\varepsilon_{00}\rangle+|1\rangle|\varepsilon_{01}\rangle\right)+\frac{1}{\sqrt{2}}\left(|0\rangle|\varepsilon_{10}\rangle+|1\rangle|\varepsilon_{11}\rangle\right) \\ =|+\rangle\frac{|\varepsilon_{00}\rangle+|\varepsilon_{01}\rangle+|\varepsilon_{10}\rangle+|\varepsilon_{11}\rangle}{2}+|-\rangle\frac{|\varepsilon_{00}\rangle-|\varepsilon_{01}\rangle+|\varepsilon_{10}\rangle-|\varepsilon_{11}\rangle}{2} \\ \text{=}|+\rangle|\varepsilon_{++}\rangle+|-\rangle|\varepsilon_{+-}\rangle\end{matrix}$$

$$\begin{matrix} U|-\rangle|\varepsilon\rangle=\frac{1}{\sqrt{2}}\left(|0\rangle|\varepsilon_{00}\rangle+|1\rangle|\varepsilon_{01}\rangle\right)-\frac{1}{\sqrt{2}}\left(|0\rangle|\varepsilon_{10}\rangle+|1\rangle|\varepsilon_{11}\rangle\right) \\ =|+\rangle\frac{|\varepsilon_{00}\rangle+|\varepsilon_{01}\rangle-|\varepsilon_{10}\rangle-|\varepsilon_{11}\rangle}{2}+|-\rangle\frac{|\varepsilon_{00}\rangle-|\varepsilon_{01}\rangle-|\varepsilon_{10}\rangle+|\varepsilon_{11}\rangle}{2} \\ \text{=}|+\rangle|\varepsilon_{-+}\rangle+|-\rangle|\varepsilon_{--}\rangle\end{matrix}$$

In error-check, these parameters should be constrained by the error rates of X basis and Z basis,

$$\begin{matrix} e_{z}=\langle\varepsilon_{01}|\varepsilon_{01}\rangle=\langle\varepsilon_{10}|\varepsilon_{10}\rangle\\ e_{x}=\langle\varepsilon_{+-}|\varepsilon_{+-}\rangle=\langle\varepsilon_{-+}|\varepsilon_{-+}\rangle\\ =\frac{1-\mathrm{Re}(\langle\varepsilon_{00}|\varepsilon_{11}\rangle+\langle\varepsilon_{00}|\varepsilon_{01}\rangle+\langle\varepsilon_{01}|\varepsilon_{10}\rangle+\langle\varepsilon_{10}|\varepsilon_{11}\rangle)}{2} \\ =\frac{1-\mathrm{Re}(\langle\varepsilon_{00}|\varepsilon_{11}\rangle-\langle\varepsilon_{00}|\varepsilon_{01}\rangle+\langle\varepsilon_{01}|\varepsilon_{10}\rangle-\langle\varepsilon_{10}|\varepsilon_{11}\rangle)}{2} \\ =\frac{1-\mathrm{Re}(\langle\varepsilon_{00}|\varepsilon_{11}\rangle+\langle\varepsilon_{01}|\varepsilon_{10}\rangle)}{2} \end{matrix}$$

It is easy to see that $S(\rho^{ABE})$ is monotonically decreasing with $\Delta_{1}$ and $\Delta_{2}$. Therefore, it takes its maximum at $\Delta_{1}=0$ and $\Delta_{2}=1-2e_{x}-2e_{z}$. Thus,

$$S(\rho^{ABE})=1+h(\xi)$$

where $\xi=\left(1-\sqrt{(1-2p)^{2}+(1-2e_{x}-2e_{z})^{2}[1-(1-2p)^{2}]}\right)/2$ and $h(x)=-x\log_{2}x-(1-x)\log_{2}(1-x)$ is the binary Shannon entropy. This attack satisfies the following equation:

$$\begin{matrix} \langle\varepsilon_{00}|\varepsilon_{01}\rangle=\langle\varepsilon_{11}|\varepsilon_{10}\rangle=0 \\ \langle\varepsilon_{10}|\varepsilon_{01}\rangle=e_{z} \\ \langle\varepsilon_{00}|\varepsilon_{11}\rangle=1-2e_{x}-e_{z} \end{matrix}$$

The maximum of mutual information between Alice and Eve is $I(A:E)\leq h(\xi)$.

References

[1] Jozsa, R. & Schlienz, J. Distinguishability of states and von neumann entropy. *Physical*

*Review A* **62**, 012301 (2000).

1. ^1^State Key Laboratory of Low-Dimensional Quantum Physics and Department of Physics, Tsinghua University, Beijing 100084, China; ^2^School of Information and Technology, Tsinghua University, Beijing, 100084, China; ^3^School of Electronic and Information Engineering, Beihang University, Beijing, 100191, China; ^4^Beijing National Research Center for Information Science and Technology, Beijing, 100084, China; ^5^Innovative Center of Quantum Matter, Beijing,100084, China; 6. Beijing Academy of Quantum Information Science, Beijing, 100193, China.

   *These authors contributed equally to this work.

   †Correspondence: LG Yin, E-mail: yinlg@tsinghua.edu.cn; GL Long, E-mail: gllong@tsinghua.edu.cn [↑](#footnote-ref-1)
